# Therapeutic educational robot enhancing social interactions in the management of obesity

**DOI:** 10.3389/frobt.2022.895039

**Published:** 2022-08-17

**Authors:** Enrico Prosperi, Giada Guidi, Christian Napoli, Lucio Gnessi, Luca Iocchi

**Affiliations:** ^1^ Società Italiana di Educazione Terapeutica, Roma, Italy; ^2^ Dip. di Ingegneria Informatica Automatica e Gestionale, Sapienza Università di Roma, Roma, Italy; ^3^ Dip. di Medicina Sperimentale, Sapienza Università di Roma, Roma, Italy

**Keywords:** social assistive robotics (SAR), therapeutic education, obesity, human-robot interaction, educational robot

## Abstract

Obesity is a chronic multifactorial pathology determined by many factors, including incorrect eating habits and a low level of physical activity. There is an urgent need to promote a persistent change in lifestyle in obese subjects, but very few individuals maintain long-term results achieved after diet therapies. Therapeutic Education (TE) has taken over an important role as a multidisciplinary intervention aimed at improving lifestyle and at acquiring new skills for the management of the disease. However, only a small portion of patients can maintain participation in such programs and fully benefit from them. Assistive technologies, and in particular assistive social robots, are powerful tools to boost independence and improve participation in educational activities. The aim of the research work described in this article is to evaluate the effect of employing a social robot as a therapeutic educational robot helping the expert therapist in the education activity. This article describes the implementation, deployment, and evaluation of a social educational robot used as a TE assistant. Although we cannot provide statistically significant results due to the limited number of people involved in the experimental protocol, all experimental results show a positive trend, indicating that the robot can enhance the social interactions between the patients and the therapist and among the patients, thus bringing to better overall results of the TE sessions, measured with standard tests for obesity management.

## 1 Introduction

Obesity is a chronic multifactorial pathology determined by genetic, psychological, social, and environmental factors, incorrect eating habits and a low level of physical activity (Rubino et al., 2020; Albury and Strain, 2020). Very few individuals maintain long-term results achieved after diet therapy (Hall and Kahan, 2018; Ge et al., 2020). There are several factors that hinder the maintenance of weight loss: environmental, emotional, biological, behavioral, cognitive, and eating disorders often characterized by binge eating and loss of control (Gupta, 2014; Evert and Franz, 2017). In recent years, therefore, there has been an urgent need to promote a persistent change in lifestyle in obese subjects, to improve adherence to treatment, and to consolidate the results obtained over time. In literature, lifestyle modification interventions in obese patients are generally focused on nutrition, exercise, and behavioral strategies and require a multidimensional and multidisciplinary approach.

In recent years, Therapeutic Education (TE) has taken over an important role in the treatment of many chronic diseases. TE is “a continuous, patient-centered process that aims to help patients and families to better understand the disease and its treatment” (Report of a World Health Organization (WHO), Regional Office for Europe, 1998). The main objective of TE is to improve management of the disease by the patient and thus reduce morbidity or the onset of certain complications. One of the secondary objectives is economic: a reduction in the need for treatment, which may lead to reduced direct or indirect costs as already shown in asthma or diabetes.

As far as obesity is concerned, TE is configured as a multidisciplinary intervention aimed at improving lifestyle and at acquiring new skills necessary for the management of the disease. In order to increase adherence to treatment and patient knowledge and skills, in previous years the group of the High Specialization Center for the Cure of obesity of the Sapienza University of Rome (CASCO) has implemented a specific program of TE for obesity and binge eating disorders consisting in educational activities carried out by a medical tutor specialist in clinical psychology (Donini et al., 2014). The interdisciplinary program is based on group therapies including physical activity, nutritional education and cognitive-behavioral techniques (Piacentino et al., 2016). However, although the vast majority of people with chronic disease who had attended education programs considered them to be helpful, only a small portion maintains participation in any such programs. Analyzing the real motivations that bring to leave the educational program is very important to improve adherence to the program in the long term. Some motivations reported by patients leaving the programs are often related to work constraints, lack of time, etc. But our conjecture is that deeper motivations are not made explicit (possibly unconsciously) by the patients.

Assistive technologies, when designed and implemented appropriately, are powerful tools to boost independence and improve participation.^1^ Social robots exploit the physical presence of a robot for an effective social interaction with people aiming at improving some aspect of human well-being.

In this article, we report the development and deployment of a social robot helping expert therapists in TE for obesity management. The presence of a social robot in the TE aims at helping obese patients with problems, such as attention deficit disorder and impulsivity symptoms, in improving the effects and the benefits of TE. More specifically, we expect the robot to improve patients’ motivation, engagement, enjoyment, and adherence to the TE, thus increasing the effectiveness of the educational content provided by the therapist, leading to overall better performance of the therapy.

**FIGURE 1:**
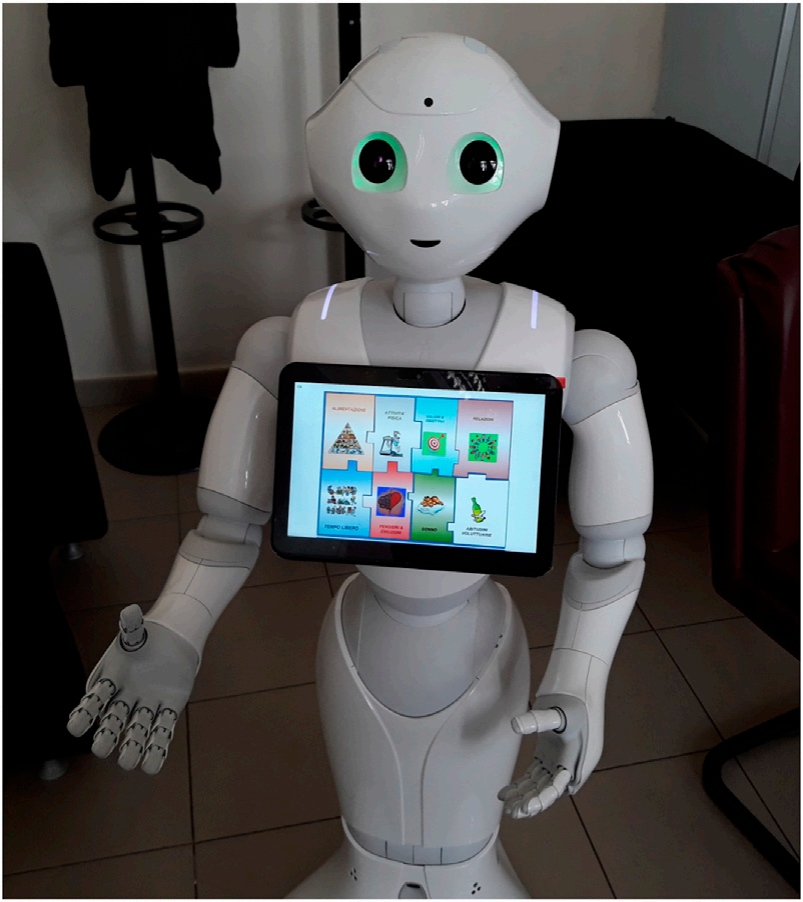
TERESA social robot.

The social robot has been named TERESA (acronym of “Therapeutic Educational Robot Enhancing Social interActions”) allowing patients to refer the robot by its name, increasing its acceptability. More specifically, we developed software components for social and educational interaction on top of the human-like robot SoftBank Pepper,^2^ shown in Figure 1.

The social robot TERESA implements a Therapeutic Educational social robot able to enhance the social interactions between the patients and the carer and among the patients. The specific goals of the project are to devise and validate a methodology to deploy social robots in TE and to measure effectiveness in patient participation in programs for a lifestyle change, by increasing their motivation, commitment, and fun and reducing their anxiety, negative moods, and embarrassment. To assess the achievement of these objectives, we used the methodology of randomized clinical trials, comparing obese patients undergoing classical TE sessions versus similar TE sessions performed with the support of the TERESA social robot. It is important to notice that the results described in this article concerned real TE sessions with the robot (not laboratory experiments). The performance metrics used to evaluate the effectiveness of the TE are well-known metrics used to assess obesity clinical status.

The results show an overall positive trend of the TE sessions helped with the social robot. Although the number of patients in the experimental group using the robot is not high enough to confirm the statistical relevance of the program, we believe that the results are very promising and the methodology and tools described in this article can be beneficial also for similar initiatives of using therapeutic educational robots in other fields.

The article is organized as follows. Section 2 describes the related work in TE for obesity management and social assistive robotics, highlighting the novelty of using a social robot in this field. Section 3 describes the functionalities of TERESA robot, while Section 4 presents the implementation of the TE sessions with the robot. The results of the TE sessions with the robot, in comparison with classical TE, are discussed in Section 5. Conclusions and future work are finally discussed in Section 7.

## 2 Related work

A recent review by World Health Organization (2011), analyzed the factors that seem to favor greater adherence to lifestyle-change programs in the treatment of obesity and every possible obstacles. Among the causes that hinder a proper participation in programs for lifestyle change, the authors have found: low levels of knowledge and awareness about obesity and eating disorders, a lack of motivation and a lack of level of fun during the course of treatment, high levels of anxiety, high levels of embarrassment.

Appropriate goals of weight management should include weight loss, maintenance and prevention of weight regain (Yumuk et al., 2015). Management of co-morbidities and improving quality of life are also included in treatment aims. Balanced hypocaloric diets and aerobic training are the optimal mode for reducing fat mass. Cognitive behavioural therapy directly addresses behaviours that require change for successful weight loss and maintenance. Pharmacotherapy can help to maintain compliance and ameliorate obesity-related health risks. Surgery is the most effective treatment for morbid obesity in terms of long-term weight loss. With the only exception of bariatric surgery, the long term effect on weight loss of these interventions is poor and the regain of body weight is almost the rule. As far as the non-surgical intervention is concerned a multidimensional multidisciplinary approach including nutritional therapy, psycho-educational classes and physical reconditioning/motor rehabilitation is the only effective intervention in the long-term outcome of obesity with regard to weight loss and control of weight regain (Donini et al., 2014).

In order to improve acceptability, engagement, and effectiveness of educational programs, technological tools, and in particular social robots, have been effectively used for assisting caregivers and patients in TE with the goal of enhancing its effectiveness (e.g., Robinson et al., 2014; Ienca et al., 2016).

In many successful experiments in these fields, it is argued that the employment of social robots in educational and health-care activities provide for improved engagement, faster learning, higher commitment, and better compliance (e.g., Valentí Soler et al., 2015; Breazeal et al., 2016; Dawe et al., 2019).

In recent years, social robots have been used in health-related domains including therapeutic interventions for children with autism, chronic disease management, health education, patient advocacy, or as a new kind of tele-medicine interface. Intriguingly, there are no reported direct application of social robots in the obesity management setting. On the contrary, literature describing experiments and results of application of social robots in education and health-care with adults and children is available (Shibata and Wada, 2011). Robots have been successfully used to assist patients with Autism (Scassellati et al., 2012), mounting evidence pointed to a possible significant association between Attention Deficit Hyperactivity Disorder including autism and certain somatic conditions, including obesity. Meta-analytic evidence confirms this association, regardless of possible confounding factors such as psychiatric comorbidities (Cortese and Tessari, 2017). Positive impacts of social robots in improving management and education in children with diabetes are reported by several works (Alotaibi and Choudhury, 2015). Although several existing studies confirm promising results of the use of social robots to improve effectiveness of therapeutic treatments, the technology is still not fully consolidated, the user studies are sometimes limited, the impact in practice is quite small, and more extensive experimental campaigns are necessary. In particular, there are no extensive studies in which experts in TE for obesity management and experts in social robotics have worked together and measured performance on a real user study.

Many studies have been conducted regarding the implementation of social robots for medical and healthcare applications. In Ljungblad et al. (2012) the authors report one of the first implementation on semi-public hospitals of social robots. In this study, a robot was implemented for a short period of time to transport clinical samples between different wards. During this experiment, several questionnaires were proposed to the clinical staff and the patients in order to assess their perception and sentiment with respect to the robot. A longer term study is reported in Hebesberger et al. (2017) where social robots are used at elderly care facilities in order to improve the quality of life of adults affected by different kinds of dementia and severe multimorbidity. This latter study also determined several guidelines for social robotics that can help to improve the end-user experience depending on the target age and mental health. The authors of Burgess et al. (2017) offer a review of Robotics for surgeon assistance. While the authors highlight that the cost of such robotic devices is one of the main limit for this kind of applications, their study also analyze and determine a set of policies for the implementation of robots in hospital environments. Generally mobile robots have been used into hospitals for internal delivery services (Jeon et al., 2017), therefore in a limited social context.

Although assistive social robots have been used in several psychological applications, applications to psychotherapy to support patients’ groups is still under study (Eichenberg et al., 2019).

The novelty of the research reported in this article is in the development, deployment and evaluation of a social educational robot as an assistant in TE sessions. More specifically, we report some design choices, implementation details, and experimental results confirming a positive trend when using the social robot to improve performance of TE sessions.

## 3 TERESA robot functionalities

TERESA is an educational social robot equipped with HRI functionalities allowing for a suitable and effective interaction with users, using a semi-autonomous mode supervised by an expert of the domain. More specifically, a set of interaction behaviors are implemented before each therapeutic meeting containing educational content useful for the patients. Therapists know in advance the topics of each meeting and the typical reactions of patients, so they can predict which kind of help TERESA could give during a session. During the actual meeting, a domain expert person collaborating with the principal therapist is supervising the robot, activating the proper interaction at the right time. In other words, TERESA is a semi-autonomous robot that is able to autonomously deliver educational content and perform educational interactions with patients, under the temporal supervision of a domain expert. This modality is necessary since the robot is not able to understand when it is the right time to intervene on a specific topic during the therapeutic session, thus making sure that it will not disturb the normal therapeutic activities.

**FIGURE 2:**
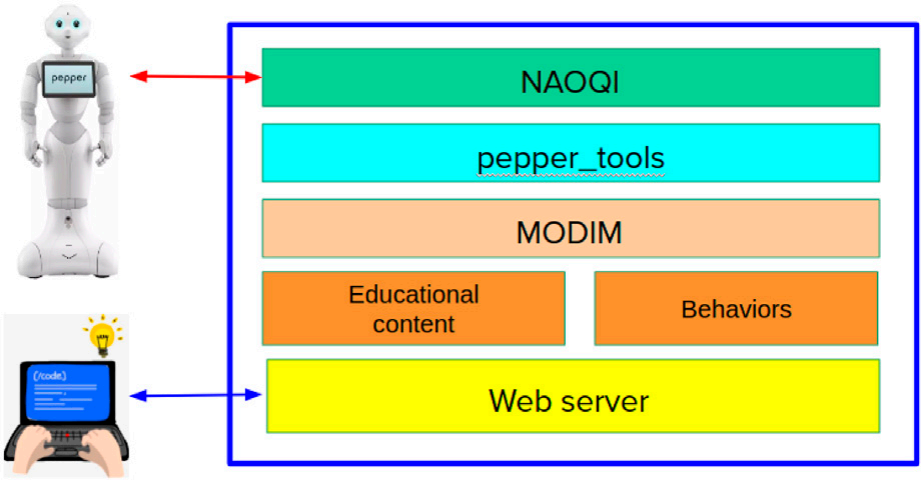
TERESA software architecture illustrating the different software layers used in the application.

TERESA software architecture is illustrated in Figure 2. All the software is running on the local CPU of the robot. The therapist user can interacting with the robot via a web browser and WiFi connection to enter educational content and control the interaction behaviors. Using a web-based interface increases usability, removing the need of installation and use of specific software by the therapist.

The web based User Interface (UI) has been designed to allow domain expert users to easily produce educational content for the robot and to supervise its deployment. This UI is based on the MODIM^3^ (Multi-MOdal Interaction Manager) library (Ferrarelli et al., 2017), which has been specifically designed for easy-to-use creation of interactions by users not expert in robotics. MODIM is also in charge of interfacing with *pepper_tools* ^4^ library containing the implementation of a set of robot functionalities and with the NAOQI system that is the low-level controller of the robot.

This architecture is implemented through multiple processes exchanging information through TCP client/server protocols. More specifically, NAOQI is a middleware running a server that accepts clients sending requests to interact with the robot’s devices (sensors and actuators). *pepper_tools* is a front-end providing high-level functions to enable and use robot’s basic functionalities though Python functions, hiding the complexity of NAOQI and Pepper specific implementations. MODIM is also running a server managing the HRI interfaces of the robot, i.e., tablet, speakers, microphones, and gestures. The web server is an HTTP server that accepts connections from web browsers and provides HTML/JavaScript code connected with MODIM server through websockets.

The behaviors are Python scripts using MODIM and *pepper_tools* high-level functions to implement robot interactions with the patients, while the educational content is a set of resources (video, audio, images. slides) that are used within the interaction behaviors.

During an educational meeting, the assistant therapist uses a web interface to enable the pre-defined behaviors when it is the right time to do it. For example, with a click on the web interface, the therapist can activate a predefined interaction behavior (using the JavaScript/websocket connection). The interaction behavior activates robot functionalities through TCP connection with MODIM server, which uses *pepper_tools* high-level Python functions, that are client modules of NAOQI server managing the robot devices. This cascade of client/server connections makes the system very modular and easy to manage and allows for high-level abstractions. Since all the processes are running in the same machine on the robot, these TCP connections introduce a minimal delay that does not affect effectiveness of the interactions.

It is important to observe that, except for the specific content of the educational material and the behaviors, the components of this system architecture are domain-independent, thus allowing to use the system for different types of educational activities and interactions. These components (except for NAOQI) are open-source and multi-platform, thus they can be used on different robotic platforms, for example, on robots developed with ROS^5^ (Robot Operating System).

## 4 Implementation of therapeutic sessions with TERESA robot

The project involves patients selected at the CASCO Center of the Department of Experimental Medicine, Sapienza University of Rome. Before being included in the groups, all patients underwent an individual visit (educational diagnosis) during which, among other things, they were asked about the history of their weight and diets, what they knew about obesity, why, according to them, they had gained weight (naive theory), and their expectations and needs. Patients also completed the following tests: Binge Eating Scale (BES), Symptom Checklist-90R (SCL90R), Short Form – 36 Health Survey (SF-36). The TE intervention is defined as Choice Education and Awareness, because it is based on third generation cognitive behavioral approaches.

Each TE session is organized in 8 meetings, lasting 2 hours every 2 weeks. The group of participants to each therapy is relatively small, between 8 and 10 patients. The therapy is led by a principal physician, and a co-therapist (psychologist or psychotherapist). Other specialists are invited in some of the meetings to address specific topics. In particular, the principal therapist is responsible for providing educational content to patients and for guiding discussions and interactions between patients and physicians and between patients themselves. As already mentioned, the commitment of the patients in actively attending the entire educational process is very important.

Each educational meeting deals with a different topic: knowledge of physiological sensations of hunger and satiety, mindful eating, the food pyramid, physical activity, management of stress and emotions, management of risk situations, relapse prevention. Within each meeting, specialists educate patients starting from the knowledge they have of the topic discussed in the session in a very experiential way. For example, for the theme of nutrition, a pyramid is built on the ground and plasticized foods are shown. Patients are then asked to arrange the foods, thus building what they think is the correct food pyramid of the Mediterranean diet and, once built, the correct one is shown. We then discuss macronutrients (carbohydrates, proteins and lipids).

In addition to provide content about these topics, the therapists involve all the patients to actively participate in the educational activities. At the end of each meeting, participants are given homeworks and are asked to experiment with the techniques or strategies they have been taught (mindfulness exercises, filling in an emotional diary, etc.) until the next meeting. At the beginning of the next meeting, the therapist asks what the participants remember from the previous meeting and what they experienced, enabling a discussion between therapists and patients and among patients about important educational content.

In the work reported in this article, we introduced a novel element in the TE sessions, which is the social robot TERESA. In this setting, therefore, the therapy has exactly the same educational content that is still given and organized by the principal therapist, helped by the co-therapist and other specialists when needed, while another member of the physician staff is responsible for the high-level supervision of the robot.

Consequently, the difference between the experimental groups (performing the TE sessions with the social robot) and the control groups (performing the TE sessions without the social robot), on which the results reported in the next section are based, is given only by the presence of the social robot and by its interaction as a mediator between the therapists and the patients.

From the educational viewpoint, TERESA is used to improve the dialogue and the interaction between the therapist and the patients. To this end, contents inserted in the robot have a lower level of depth and knowledge than the ones provided by the principal therapist, so as not to confuse patients about the role of the main physician compared to the robot assistant.

The interactions implemented in the TERESA robot support the topics described by the principal physician and are typically provided in the form of summaries of what has been said previously, examples of applications and questions. TERESA often refers to “her friends” to illustrate examples useful for discussion during the therapy session. For example, the robot helps to ease the initial tension, by initiating to describe some experiences as expected by the therapist, thus providing guidelines to be followed by the patients. In case of a patient impasse, the robot intervenes by telling something about the previous meeting and what it has done; on other occasions, it asks questions in quiz mode and invites patients to answer as in a game, to verify the understanding of what has been talked about and possibly correct wrong answers, in an acceptable way for the patients. It is important to underline here that the same behavior made by a therapist would not have the same impact on the patients. In fact, the patients do not feel that TERESA robot is evaluating in any way their behavior (both inside and outside the meeting). Moreover, some content inserted in the robot is deliberately wrong to allow the physician to comment about wrong habits or behaviors to all the patients. In this way, the patients tends to accept the robot as a mediator between them and the therapists.

**FIGURE 3:**
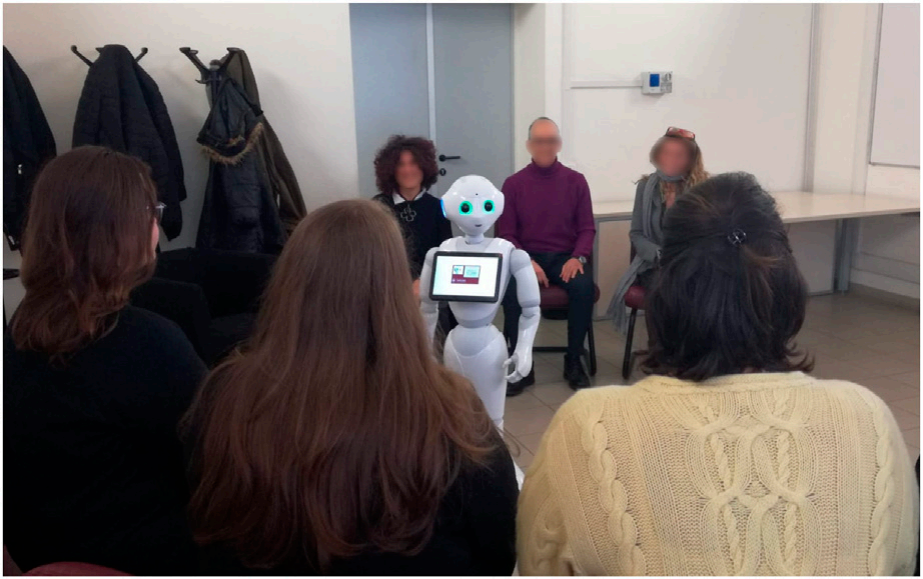
TE session with TERESA social robot.

In Figure 1, TERESA shows and explains lifestyle concepts through the use of images on the tablet and voice. Figure 3 shows a group integration in which TERESA invites the participants to express their opinion on some issues addressed during the meeting.

As already mentioned, the robot supervisor has the role of guiding the timing of the robot interactions, using a simple web interface with a few buttons. The assistant physician (expert in the obesity therapy, but not expert in robotics and artificial intelligence) decides when the time is right for the robot to step in and deliver the previously developed educational content, thus synchronizing the robot interactions with the activity of the principal physician. After the robot supervisor starts the interaction, this is carried out by the robot autonomously.

It is important to highlight that the supervision of the robot is not explicitly hidden from patients. Indeed the medical assistant is present in the room where the therapy session takes place and does not hide the fact that s/he interacts with the robot through a laptop. Nonetheless, patients tend to identify the robot as an “intelligent” autonomous assistant effectively supporting the primary physician.

## 5 Evaluation methodology and results

At the end of the TE session, a performance evaluation is carried out in order to assess the effectiveness of the educational activity. Three types of measures are relevant in this project: 1) measures related to medical indicators, already defined and used to evaluate the effectiveness of TE in these contexts; 2) measures related to indicators of human-robot interaction and acceptability of social robots, to evaluate the use of the robot in such therapeutic sessions; 3) measures to predict the outcome of the TE session from data acquired during the sessions. For the medical indicators, the results of the experimental group performing TE sessions with TERESA robot are compared with the results of a control group obtained during TE sessions without the use of the social robot.

Metrics used as performance metrics and results about medical indicators and HRI performance are described in the next sections. For the prediction analysis, we describe the proposed evaluation methodology and techniques that can be used to develop a solution, while results are left as future work.

### 5.1 Therapeutic analysis

The following medical indicators have been used to assess the quality of the TE sessions, both with and without the use of a social robot.

1) Body mass index (BMI) is a person’s weight in kilograms divided by the square of height in meters. BMI is an inexpensive and easy screening method for weight category—underweight, healthy weight, overweight, and obesity. However it is only a surrogate measure of body fatness. Obesity is defined as a body mass index (BMI) equal to or greater than 30 kg/m^2^. According to the WHO classification, a BMI of 30.0–34.9 corresponds to class I obesity, a BMI of 35.0–39.9 corresponds to class II obesity, and a BMI ≥40.0 corresponds to class III obesity (i.e., morbid or severe obesity).
2) Binge Eating Scale (BES) Gormally et al. (1982) is a 16-items self-administered questionnaire. For each item, patients have to choose between three or four sentences the one that best describes the way they feel and their behavior. Each answer corresponds to a different score. The total score is obtained from the sum of the scores of the single items. The questionnaire assesses the psychological characteristics associated with binge eating, but does not provide an estimate of the number of binge episodes. The score ranges from 0 to 46 and by using a cut-off of 17, the questionnaire can be used as a screening tool for BED in obese patients and is taken as comparable to the SCID-I, the gold standard for the diagnosis of Binge Eating Dirorder (BED). In particular, a score lower than 17 indicates non-binging, a score of 17–26 indicates moderate binging, and a score of 27 or higher indicates severe binging.
3) Symptom Checklist-90-Revised (SCL-90-R) Derogatis (1992) encloses 90 items and assesses the psychological status and the symptoms of psychopathology of medical and psychiatric patients, as well as of healthy individuals. Subjects are asked to describe how much they have suffered from each of the symptoms covered by the 90 items in the previous 7 days. Responses are given on a 5-point Likert scale of distress ranging from 0 (“not at all”) to 4 (“extremely”). This allows to derive a general symptom level, known as the global severity index (GSI), and nine subscales: somatization, obsessive-compulsive, interpersonal sensitivity, depression, anxiety, anger-hostility, phobic anxiety, paranoid ideation, and psychoticism. Higher GSI scores indicate a greater overall psychopathological disturbance; higher subscale scores indicate a greater intensity of disturbance in the examined areas. The cut-off for the GSI is 0.566, as indicated by the existing literature (Schauenburg and Strack, 1998; Schauenburg and Strack, 1999): scores equal to or above 0.566 are considered to be indicative of “dysfunctional” subjects (i.e., distressed subjects showing symptoms of somatic and psychological suffering, whose severity lies “within a dysfunctional range”), as opposed to “functional” subjects (healthy subjects, whose symptom severity lies “within a functional range”). “Dysfunctional” subjects have a high probability of psychiatric disorders. Scores equal to or above 1, both for the GSI and the nine subscales, are considered to be indicative of “psychiatric” subjects, who have clinically evident psychiatric disorders. In particular, we analyzed the sub-scales SCL90R-DEP, SCL90R-ANX, SCL90R-HOST, that evaluates respectively the level of depression, anxiety, and anger-hostility. These sub-scales are particularly important for obesity management.
4) Short Form (36) Health Survey (SF36) Taylor and Sirois (2012). It includes 36 items that measure patients’ health status. It is commonly used in health economics to determine the cost-effectiveness of a health treatment. It takes into account two major domains, which contribute to the Quality of Life (QoL): physical and psychological health. Eight subscales can be derived and they represent the weighted sums of the questions in their section: vitality/fatigue, physical functioning, role limitations due to physical problems, role limitations due to emotional problems, general health perceptions, bodily pain, social functioning, general mental health. Each subscale is directly transformed into a 0–100 subscale on the assumption that each question carries equal weight. Moreover, a summary of physical QoL (Physical Component Summary, PCS) and psychological QoL (Mental Component Summary, MCS) can be obtained by working out the average of all of the physically relevant items and of all of the emotionally relevant items mentioned above. For each component summary the mean score is 50 with a standard deviation (SD) of 10. The lower the score, the more severe the disability; the higher the score, the less severe the disability (i.e., a score of zero is equivalent to maximum disability, meaning minimum QoL in that area, and a score of 100 is equivalent to no disability, meaning maximum QoL in that area).

### 5.2 Comparative analysis

In this section, we describe the evaluation methodology to assess the effectiveness of the use of a social robot in the therapeutic educational activity. This methodology is based on comparing the performance indicators described in the previous section between an experimental group (using the robot) and a control group (not using the robot). Given the organization of the TE sessions, a between-subject experimental modality has been used. All the TE sessions, for both control and experimental groups, contain the same educational content and are given by the same therapists (co-authors of this article). At this moment, we compare the performance of a large baseline control group of patients who participated in TE sessions in the last years without the presence of TERESA educational robot with a small experimental group (16 patients) who participated to TE sessions with TERESA robot, since March 2019. The small number of people in the experimental group does not allow to assess statistical significance of the results, but just to evaluate the trend that is overall positive and very promising.

**FIGURE 4:**
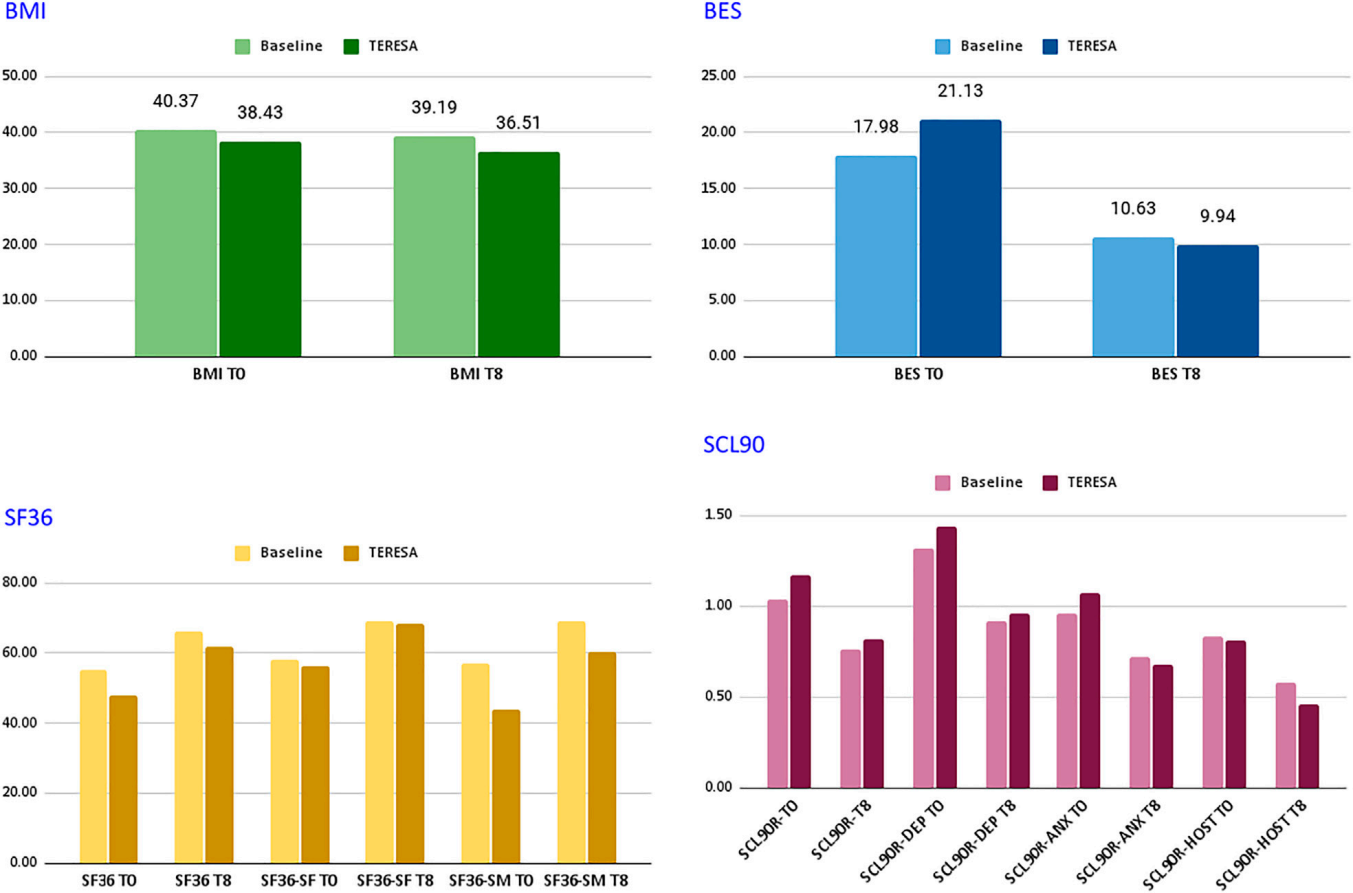
Comparison between baseline and TERESA groups.

Figure 4 and Table 1 report the average scores obtained by the two groups of patients on the different indicators. As shown in the table, although starting from different values of BMIs, an improvement in the experimental group has been observed (from −3.3% to −5.0%). Guidelines on obesity control define clinically significant weight loss as at least a 5% reduction in weight from the baseline level and usually most obese patients lose only modest weight with non-pharmacological interventions alone. The results obtained by the experimental group in a short period of time are in-line with such guidelines and thus very encouraging.

**TABLE 1:** Comparison between baseline and TERESA groups. All indicators (in both control and experimental groups) denote improvements in the specific metric.

Test	Baseline—Control group	TERESA—Experimental group
Participants	259	16
BMI	40.40 → 39.06 (−3.3%)	38.43 → 36.51 (−5.0%)
BES	17.96 → 10.61 (−40.9%)	21.13 → 9.94 (−53.0%)
SF36	55.30 → 66.03 (+19.4%)	48.08 → 61.82 (+28.6%)
SF36-SF	58.05 → 68.92 (+18.7%)	56.46 → 68.23 (+20.8%)
SF36-SM	57.03 → 68.93 (+20.9%)	43.73 → 60.45 (+38.2%)
SCL90R	1.05 → 0.77 (−26.8%)	1.17 → 0.82 (−30.1%)
SCL90R-DEP	1.32 → 0.92 (−30.3%)	1.44 → 0.96 (−33.3%)
SCL90R-ANX	0.96 → 0.72 (−24.6%)	1.07 → 0.68 (−36.8%)
SCL90R-HOST	0.83 → 0.58 (−29.9%)	0.81 → 0.46 (−43.6%)

Both groups showed improvements in the BES indicator: from a score within 17–26, indicating moderate binging to a score < 17 which indicates non-binging. Again the improvement is more evident in the experimental group (from −40.9% to −53.0%). This result is important as the BES constitutes a predictive index for the efficacy of the treatment and can prevent the patient to undergo a bariatric surgical intervention.

A general improvement in the SF36 is also noticed, although in the experimental group both the total score and that of the psychological QoL started from a value lower than 50.

Finally, in the SCL 90 R the Global Severity Index (GSI) and sub-scale depression decreased below 1 in both groups, which is considered to be the limit for psychiatric disorders. This result is important as depression is common in people with Type-II and Type-III obesity and both depression and obesity are associated with an increase in cardiovascular diseases. Consequently, reduction of this indicator (again higher in the experimental group) is an important success factor for obesity therapies.

### 5.3 HRI analysis

The Human-Robot Interaction components of TERESA educational robot have been evaluated using the well-known methodology based on the GodSpeed questionnaire (Bartneck et al., 2009). The objective of this evaluation was to assess the acceptability of an educational robot during TE sessions both from the patient and from the therapist perspective. The 16 patients who attended the TERESA groups and 9 therapists that were present during the TE sessions were involved in this study. The questionnaires were filled by both patients and therapists at the end of the TE sessions.

The results reported in Table 2, average over a 5-point Likert scale, show good performance of the robot according to the HRI parameters identified in the Godspeed questionnaire. More specifically, scores for likeability, perceived intelligence and perceived safety are very high, showing a very high acceptability of the robot. On the other hand, anthropomorphism and animacy have a medium-high score, depending on the actual nature of the robot, properly designed to avoid uncanny valley effect, and the type of task not allowing large mobility of the robot during the therapies.

**TABLE 2:** Results of Godspeed questionnaire: average over a 5-points Likert scale.

	Overall	Patients	Therapists
Anthropomorphism	3.514	3.508	3.523
Animacy	3.645	3.645	3.644
Likeability	4.692	4.694	4.689
Perceived Intelligence	4.262	4.323	4.178
Perceived Safety	4.548	4.486	4.630

Using a *t*-test, we have compared the results of the two groups of users: patients and therapists. All *p*-values for all the answers are above 0.1, thus indicating that there has been no significant difference between the evaluation made by patients and therapists. Thus, although with low confidence due to the limited number of users, we can say that patients and therapists evaluate the HRI abilities of the robot in the same way. This confirms that the robot is a good tool for both the groups.

## 6 On-going work on predictive analysis

When tackling with long-term therapies, such as for the treatment of eating disorders, an early prediction of the therapeutic outcomes would be a valuable asset, especially for therapy planning and adjustment in progress. In order to obtain an objective measurement of the therapy outcomes, psychometric tests are often used, therefore it would be beneficial to predict the time evolution of such tests, and in particular to obtain an accurate prediction of the obtainable final score starting from the initial scoring and a few midterm measurements.

This problem constitutes a typical time series prediction, which is a well studied task in many scientific fields and it is essential in many decision processes. On the other hand, solutions generally rely on a large amount of data, that are difficult to gather in real therapeutic scenarios.

**FIGURE 5:**
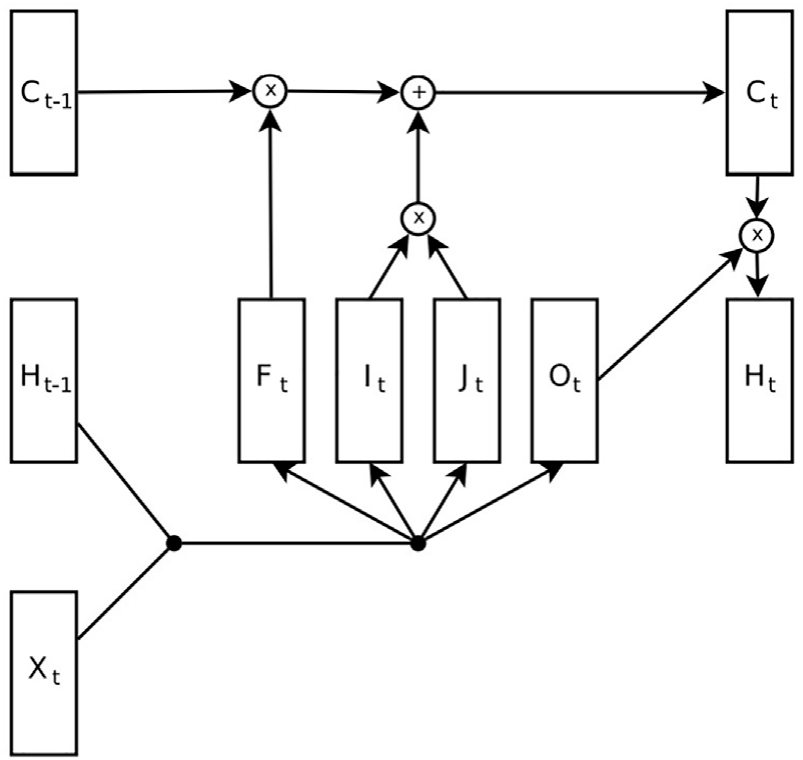
Long Short-Term Memory diagram, where C is the memory cell and it contains the state. F,I,O are respectively the forget, input and output gates. H is the hidden vector (the output of the network) that depends both on the input X and state C. Original image from Jozefowicz et al. (2015).

From the technical viewpoint, we envision the use of time dynamic neural networks, such as Recurrent Neural Networks (RNNs) or Long Short Term Memory networks (LSTM, Figure 5). In fact neural networks have been widely applied to multidimensional time series prediction, and they outperformed many model based approaches used in the past, especially for multidimensional data with nonlinear patterns (Chakraborty et al., 1992).

Also the use of multiresolution decomposition could allow us to look at the signal at different scales and to train a sub-network to model the behaviour of the signal for that specific scale. The idea of decomposing the signal to model specifically its different behaviours is not new, and it is employed also in classic model based approaches like ARIMA, where the signal is decomposed into trend, seasonal and remainder components (Hyndman and Athanasopoulos, 2018).

Many models proposed in the literature address the problem of single-step ahead prediction, meaning that the predicted samples are consecutive to the ones received as input, and the multi-steps ahead prediction is obtained running the model on the previous predictions. With this approach however the errors tend to accumulate and consequentially the performances drop when the forecast horizon increases. The proposed method, on the other hand, is designed specifically to predict samples that are multiple steps ahead in the future without relying on the intermediate predictions.

In this application, the dataset would be composed by tuples of numbers for every patient (each number represents the scoring related to a test, subtest or item). Due to the dataset topology, we devised a functional neural architecture composed by multiple LSTM encoders and dense layers (one for each band of a multiresolution decomposition). The LSTM encoders process a sequence of N steps, for each LSTM there is a dense layer that uses the encoding to predict the coefficients at that resolution level for a single step that is M steps ahead in the future. In our case the LSTM should be used for the prediction of the test score at the end of the TE, therefore giving us an index of success for the therapy. The input should be the score obtained at the beginning of the TE. In order to do that an extensive dataset is required, therefore a prolonged follow-up would allow us to collect a sufficient amount of data within a reasonable time-span to perform such kind of analysis.

The evaluation methodology presented in this section was only partially applied in the TE sessions developed so far and presentation and discussion of the results is left as future work, with an increased size of the training data. Nevertheless, preliminary results based on a few data collected at the beginning of the TE session show the feasibility of the proposed approach and the ability to learn the key features for predictive analysis.

## 7 Conclusion

In this article, we described the implementation, deployment, and evaluation of a social educational robot used as an assistant for TE sessions for obesity management. The proposed solution aims at providing the patients with a social mediator between them and the doctors and, consequently, at improving the effectiveness of the educational activity. Experimental results, in comparison with a control group not using the robot, show promising performance improvements. In addition to specific performance indicators, the social robot TERESA stimulated curiosity and participation within the group, improving interactions among patients and between patients and therapists. The robot is well accepted by both patients and therapists and future work is planned to increase the size of the experimental group to assess also the statistical significance of the improved performance.

Our research paves the way for the clinical use of assistive technology, highly promoted by the WHO, to help people with numerous disabling clinical conditions improve their quality of life and acquire self-management skills. In the particular case of obesity disease, assistive technology can help to improve adherence to the treatment, given the to achieve the WHO target of “no increase in obesity prevalence by 2025.” We envision the application of TE for obesity also to other segments of the population, such as children and adolescents, which is a very serious problem at the moment. Indeed, without substantial interventions to prevent and treat childhood obesity, the number of school-age children and adolescents living with obesity is predicted to rise from the current estimates of around 150 million worldwide to over 250 million by 2030. In this context, social robots and assistive technology may be even more effective, given the typical acquaintance with the technology of children and adolescents.

## Data Availability

The raw data supporting the conclusion of this article will be made available by the authors, without undue reservation.

## Footnotes

1 As stated, for example, in the World Report on Disability (World Health Organization, 2011).

2
https://www.softbankrobotics.com/emea/en/pepper

3
https://bitbucket.org/mtlazaro/modim/

4
https://bitbucket.org/mtlazaro/pepper_tools

5
www.ros.org
